# Corticosteroids and long-term pulmonary function after critical illness due to COVID-19– a single-center cohort study

**DOI:** 10.1186/s12890-025-03659-0

**Published:** 2025-04-26

**Authors:** Mari Roël, Anna Schandl, Sandra Jonmarker, Anders Hedman, Gisela Vogel, Eva Joelsson-Alm, Maria Cronhjort, Pernilla Darlington

**Affiliations:** 1 Department of Internal Medicine, Södersjukhuset, Stockholm, SE-118 83 Sweden; 2 Department of Clinical Science and Education, Karolinska Institutet, Södersjukhuset, Stockholm, SE-118 83 Sweden; 3 Department of Perioperative and intensive care, Södersjukhuset, Stockholm, SE-118 83 Sweden; 4 Department of Cardiology, SE-118 83 Södersjukhuset, Stockholm, Sweden; 5 Department of Clinical Sciences, Danderyd Hospital, Section of Anesthesiology and Intensive Care, Karolinska Institutet, Danderyds Sjukhus, Stockholm, SE-182 88 Sweden

**Keywords:** Corticosteroids, COVID-19, Long-term effects, Intensive care

## Abstract

**Background:**

Early in the pandemic, corticosteroids became standard treatment for patients with critical COVID-19 infections. This study aimed to investigate the possible long-term pulmonary consequences after corticosteroid treatment in patients with critical COVID-19 requiring ventilatory support.

**Methods:**

This observational single-center cohort study included patients treated for critical COVID-19 requiring ventilatory support between March 1, 2020, and August 1, 2021, with a 6-month follow-up after discharge from the intensive care unit. Corticosteroid treatment was defined according to the RECOVERY trial (6 mg dexamethasone daily or equivalent dose of another corticosteroid, initiated within eight days of hospital admittance and continued for at least one day) Pulmonary function was assessed by diffusion capacity for carbon monoxide. Health-related quality of life was measured with the questionnaire RAND-36. General linear regression was used to present mean score differences with 95% confidence intervals.

**Results:**

Among the 456 (69%) critically ill COVID-19 patients who survived at least 90 days after ICU discharge, 286 (63%) attended the follow-up six months later. The groups were balanced regarding invasive ventilation; 47% received invasive ventilation in both groups. Corticosteroid treatment was associated with a lower diffusion capacity for carbon monoxide (MSD − 8.3, 95% CI: -14.2 to -2.4) 6 months after ICU discharge (change > 10% were regarded as clinically significant). There were no differences in health-related quality of life between the groups.

**Conclusions:**

Corticosteroids might negatively impact pulmonary function after critical COVID-19. The decrease did not seem to influence health-related quality of life. Future studies are needed to confirm the results.

**Supplementary Information:**

The online version contains supplementary material available at 10.1186/s12890-025-03659-0.

## Introduction

The disease spectrum of SARS-CoV-2 infection ranges from asymptomatic disease to critical illness with acute respiratory failure requiring invasive respiratory support [1]. Critical COVID-19 is associated with a high degree of inflammation and immunomodulators have been added to the regimen. Following the press release of the RECOVERY trial in June 2020, which demonstrated reduced mortality with corticosteroid treatment, corticosteroids became the cornerstone of the anti-inflammatory regimen for critically ill patients requiring respiratory support [2–4].

Long-lasting functional impairment has been reported among intensive care unit (ICU) survivors in the first year following hospital discharge [5–8]. Survivors of severe COVID-19 show a higher proportion of impairment of diffusion capacity for carbon monoxide (DLCO) compared to predicted values and chest CT visualizes remaining abnormalities at 3-month follow-up [9]. Except for the variation in the severity of disease due to viral strains [10], there may be different causes of pulmonary sequelae between patients during different periods as treatment recommendations evolved. For example, during the latter part of the pandemic, the diagnosis of secondary bacterial pneumonia increased coinciding with the introduction of immunomodulation [11, 12].

Corticosteroid treatment in COVID-19 is given in a phase where inflammation is believed to dominate and viral replication declines [13]. The effect of corticosteroid treatment in acute respiratory distress syndrome (ARDS) is likely explained by its anti-inflammatory effect [14, 15]. Little is known about serious adverse events of corticosteroid treatment, where findings are inconsistent and difficult to interpret because of potential confounders. Therefore, this study aimed to investigate the possible long-term pulmonary consequences after corticosteroid treatment in patients with critical COVID-19 requiring ventilatory support.

## Methods

### Study subjects

This study is a prospective, single-centre cohort study including surviving patients with critical COVID-19, defined as those requiring intensive care due to respiratory failure, at a large emergency hospital in Sweden between March 1, 2020, and August 1, 2021. Critically ill adult (≥ 18 years) patients with a positive polymerase chain reaction test for COVID-19 who were treated for respiratory failure with high-flow treatment nasal oxygen (HFNO), non-invasive ventilation (NIV), and/or invasive ventilation in the ICU were eligible for inclusion in the study. Exclusion criteria were either already on corticosteroid treatment for other reasons or corticosteroid treatment not fulfilling the definition of the RECOVERY trial [13] defined as six mg dexamethasone daily or an equivalent dose of another corticosteroid, initiated within eight days of hospital admittance and continued for at least one day. Reporting of the study followed the STROBE checklist [16].

### Setting

The total number of ICU beds was 16 before the pandemic, expanding to a maximum of 60 beds during the first wave in Sweden (March-August 2020) and 33 later in the pandemic [6]. Staffing during the study period was a patient-to-nurse ratio of 1:1–2 and a patient-to-physician ratio of 1:4–6, but the proportions decreased during the peaks of the pandemic. At the turn of May/June, after the peak of the first wave, corticosteroids were introduced and had previously only been given in isolated cases. Later in the pandemic treatment with remdesivir and tocilizumab were introduced, see Supplementary Table 1.

### Data collection

All ICU survivors treated with ventilatory support due to COVID-19 were invited to attend a hospital follow-up visit and complete a questionnaire assessing their health-related quality of life (HRQL), six months after ICU discharge. Non-responders received two reminders about the follow-up.

Data were recorded from electronic medical records regarding age, sex, body mass index (BMI), smoking habits, and comorbidities i.e. diabetes mellitus, hypertension/cardiovascular disease and chronic lung disease [17]. Further data collected from medical journals were the severity of illness according to Simplified Acute Physiological Score III (SAPS 3) [18], length of ICU stay, and corticosteroid treatment.

### Outcomes

The primary outcome was pulmonary function, measured as the capacity for gas exchange, and the secondary outcome was HRQL approximately six months after ICU discharge.

To evaluate pulmonary impairment, the capacity for gas exchange, i.e. DLCO (uncorrected value, i.e. not corrected for lung volume or hemoglobin value) was measured with standard methods [19] and in accordance with reference values (Hedenström) [20, 21].

HRQL was assessed with the 36-item questionnaire RAND-36 Item Health Survey (RAND Corp). The instrument has 36 items and measures eight domains of HRQL. The different domains together with the number of items of each domain are: Physical Functioning (limitations in physical activities because of health problems), Role physical (limitations in work or daily activities), Bodily pain (limitations due to pain), General health (subjective assessment of overall health), Vitality (evaluates energy levels and fatigue), Social functioning (limitations in social activities due to physical and emotional health problems), Role emotional (limitations on work and daily activities due to emotional problems) and Mental health (assesses general mental health status) [22]. There is also one item about reported health transition and one general item about perceived health. The items are answered using Likert scales and summarized using the Likert method of summated ratings. Questionnaire responses were linearly transformed into scores between 0 and 100, where a higher score represented a higher HRQL [22, 23]. The Swedish version of RAND-36 has been validated as a reliable instrument for HRQL in the general and ICU population [24–26].

### Statistical analyses

Descriptive statistics were presented as counts (n), proportions (%), and medians with interquartile range (IQR) according to the type and distribution of data. Potential differences between groups were analyzed by Fisher´s Exact Test for categorical data and Mann-Whitney U-test for continuous variables. Multivariable linear regression models were used to estimate mean score differences (MSD) with 95% confidence intervals (CI) to assess lung function changes (DLCO) and health quality of life (RAND-36 scores) between those treated with corticosteroids and those without. The models were adjusted for presumed clinically relevant variables, age > 65 years (yes/no), sex (male/female), chronic lung disease (yes/no), tobacco smoking habits (ever/never), and length of ICU stay (continuous variable). In an additional model, SAPS 3 (as a continuous variable) and invasive ventilation (yes/no) were adjusted for. According to American Thoracic Society guidelines, clinically important differences in DLCO were set to 10% between groups [27]. For RAND-36 scores, MSDs of 5 points between groups were considered as a clinically relevant difference [23, 28]. Statistical significance was only tested for if the MSDs were clinically relevant. Missing items were accounted for with automatic calculation of the scores, according to the RAND-36 scoring protocol [22]. If more than half of the scores in one domain were missing, no result was presented. Statistical analyses were performed with Jamovi version 2.3.19 and an experienced biostatistician validated all analyses.

## Results

### Participants

**Fig. 1:**
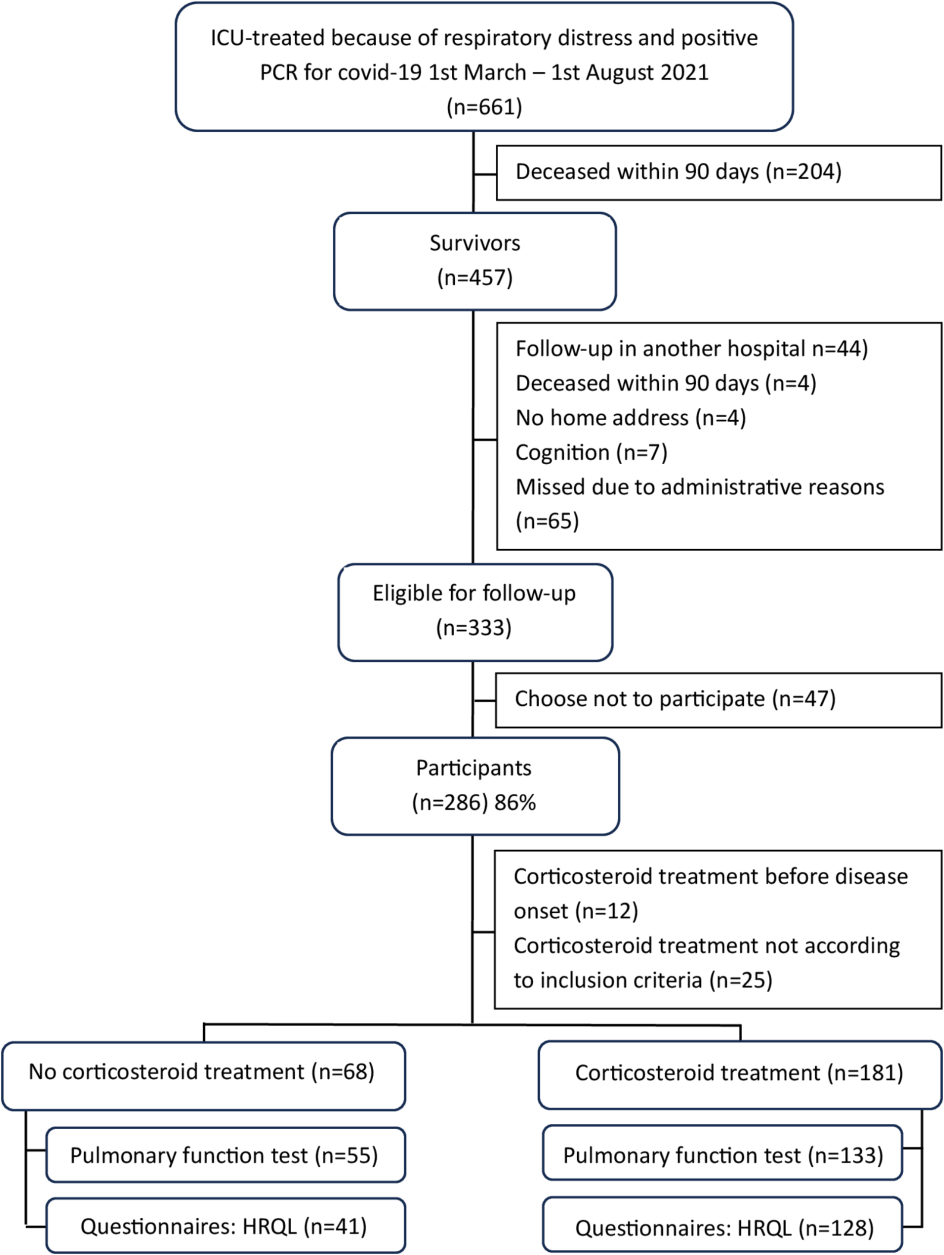
Flowchart of study inclusion, left shows patients without corticosteroid treatment and right corticosteroid treatment

During the study period, 456 (69%) critically ill COVID-19 patients survived for at least 90 days after ICU discharge, and of those 286 (63%) attended the follow-up approximately six months after ICU discharge (Fig. 1). Among these, 68/286 (24%) had not received treatment with corticosteroids and they were all survivors of the first wave. There were 181/286 (63%) patients who received treatment with corticosteroids according to inclusion criteria. The group of patients who had not received corticosteroid treatment had lower calculated mortality risk in hospital and shorter ICU stay in comparison to those treated with at least 6 mg dexamethasone or an equivalent dose daily (Table 1).

**Table 1:** Characteristics of included COVID-19 intensive care unit survivors who received corticosteroid treatment versus without

	No corticosteroids	Corticosteroids according to inclusion criteria	*p*-value
Total number, n	68	181	
Follow-up time, months Md (IQR)	5 (5–6)	6 (5–8)	< 0.01
Age, years Md (IQR)	57 (50–66)	61 (53–68)	0.06
Male n (%)	54 (79)	131 (72)	0.33
BMI, kg/m^2^ Md (IQR)	28 (25–31)	29 (26–33)	0.22
Diabetes n (%)	12 (18)	40 (22)	0.49
Hypertension/cardio-vascular disease n (%)	28 (41)	97 (54)	0.09
Chronic lung disease n (%)	10 (15)	40 (22)	0.22
Ever smoker n (%)	22 (32)	83 (46)	0.06
SAPS 3, Md (IQR)	53 (49–57)	57 (52–64)	< 0.01
Invasive ventilation n (%)	32 (47)	85 (47)	1.0
Length of ICU stay (days), Md (IQR)	8 (3–17)	10 (7–20)	< 0.01

BMI = body mass index; IQR = Interquartile range; Md = Median; SAPS = Simplified Acute Physiology Score (missing information in 4 participants in the group with no corticosteroids and 28 in the corticosteroid group)

When comparing the group without corticosteroid treatment with those who received treatment within the first wave, there was no difference in the frequency or length of invasive ventilation between the groups, the median for the whole group was 12 days with invasive ventilation.

The patients with corticosteroid treatment performed spirometry at a median time of six months and those without at five months (*p* < 0.05).

### Pulmonary outcomes

Those who received corticosteroid treatment had a statistically significant worse pulmonary outcome at 6 months post-ICU measured as diffusion capacity DLCO (MSD − 8.3, 95% CI: -14.2 to -2.4) compared to those who did not receive corticosteroids (Table 2). Similar results were seen for those with complete data regarding DLCO and SAPS 3, *n* = 162, with adjustment also for SAPS 3 and invasive ventilation (MSD − 8.0, 95% CI: -14.2 to -1.9).

**Table 2:** Mean differences (in percentages) with 95% confidence intervals (CI) and diffusion capacity (DLCO) comparing COVID-19 intensive care unit survivors who received corticosteroid treatment versus without. No corticosteroids were used as a reference

	No corticosteroids Mean scores (95%CI) *n* = 55	Corticosteroids Mean scores (95%CI) *n* = 133	Unadjusted analysis MSD (95%CI)	Adjusted analysis MSD (95%CI)
*DLCO % of predicted*	88 (83–93)	77 (74–81)	-10.9 (-17.0 to -4.7)*	-8.3 (-14.2 to -2.4)*

Abbreviations: DLCO = diffusion capacity for carbon monoxide; IQR = interquartile range. MSD = mean score difference. The models

were adjusted for age, sex, tobacco smoking, chronic lung disease and length of ICU stay. *p-value < 0.05

### Health-related quality of life

Among participants who reported HRQL data, there were no statistically significant differences between the groups (Table 3).

**Table 3:** Health-related quality of life (HRQL) comparing COVID-19 intensive care unit survivors who received corticosteroid treatment versus without, presented as mean score differences (MSD) with 95% confidence intervals (CI). No corticosteroidswere used as a reference

Health-related quality of life domains	No corticosteroids	Corticosteroids	Unadjusted analysis	Adjusted analysis
	Mean scores (95%CI)	Mean scores (95%CI)	MSD (95%CI)	MSD (95%CI)
Total number	41	128		
*Survivors*				
Physical function	66 (58–75)	65 (60–70)	-1.7 (-11.3 to 8.0)	0.8 (-8.3 to 9.8)
Role physical	43 (29–57)	46 (38–54)	2.6 (-13.3 to 18.4)	6.0 (-9.5 to 21.6)
Bodily pain	64 (55–74)	68 (63–73)	3.7 (-6.5 to 13.9)	4.8 (-5.3 to 15.0)
General health	52 (45–58)	56 (52–60)	4.3 (-3.4 to 12.0)	5.2 (-2.3 to 12.9)
Vitality	50 (43–57)	54 (49–58)	3.9 (-4.7 to 12.6)	3.8 (-4.8 to 12.4)
Social function	64 (56–73)	67 (62–72)	3.1 (-7.1 to 13.4)	4.4 (-5.6 to 14.5)
Role emotional	59 (46–72)	69 (62–76)	10.2 (-4.4 to 24.8)	12.4 (-2.1 to 26.9)
Mental health	68 (62–75)	73 (70–77)	4.9 (-2.3 to 12.0)	4.4 (-2.8 to 11.7)

The model was adjusted for age, sex, tobacco smoking and chronic lung disease

### Sensitivity analysis

There was no difference in the distribution of age, sex, comorbidities, corticosteroid treatment and type of ventilation between responders who attended follow-up (i.e. performed pulmonary function test respectively questionnaires) and non-responders (Supplementary Table 2). Those with missing information about SAPS 3 had less often received mechanical ventilation (*p* < 0.05) (Supplementary Table 3).

## Discussion

In this study, patients who did receive corticosteroid treatment had a worse diffusion capacity for gas exchange (DLCO) at 6 months follow-up, although not meeting our definition of clinically relevant difference. For health-related quality of life outcomes, there was no statistically significant difference between the groups.

We excluded patients who had been on corticosteroids before disease onset, as they received corticosteroid treatment during the virus replication phase of COVID-19 and were believed to have a less good immune response, and they consisted of a higher proportion of women and were older. We also excluded those who had not received corticosteroids according to inclusion criteria since they were judged to have received it as “rescue treatment” early in the pandemic before corticosteroids were routinely given and a confounding indication, and they had been treated with invasive ventilation to a higher extent with a longer stay in the ICU.

The corticosteroid-treated group in this study had longer ICU stay. Since corticosteroid treatment has been associated with higher frequency of ventilator-associated pneumonia (VAP) this may be a possible explanation [29]. The participants not treated with corticosteroids were all from the first pandemic wave before treatment with corticosteroids was routinely given. They seemed to have been less severely ill at ICU admission as reflected by lower SAPS 3 values, used to predict hospital mortality. This may have contributed to the better recovery for the group not treated with corticosteroids. While the benefit of corticosteroid treatment on short-term mortality is well described [3], the knowledge of long-term outcomes is sparse and uncertain. One study by Menendéz et al., showed that one year after severe COVID-19 corticosteroid treatment on one hand was associated with incomplete radiological resolution but on the other hand not with impaired DLCO [30]. However, other studies at 3 months follow-up have opposing results. A randomized controlled trial showed that corticosteroid treatment improved forced vital capacity (FVC) in hospitalized patients with COVID-19 [31], which is in line with another study also showing better FVC for patients treated with corticosteroids [32]. Forced vital capacity on the other hand does not give information about the lungs’ capacity for gas exchange, here DLCO is a more sensitive variable for parenchymal damage [33]. Furthermore, vaccination against COVID-19 in the region started with the most fragile and oldest persons first and for adults ≥ 75 at the end of March 2021 [34]. Vaccination is therefore believed to have had little impact on this cohort.

There may be different explanations for the findings of worse pulmonary outcomes in this study for those treated with corticosteroids. Corticosteroids are used to suppress inflammation and prevent lung injury and guidelines now include corticosteroids for severe community-acquired pneumonia (sCAP) as it has showed to reduce short-term mortality but little is known about long-term effects [35]. Recent research on COVID-19 has shown that even if the etiology of ARDS is the same, subgroups might have different effects of corticosteroid treatment, which might be a contributing explanation to the heterogeneity in results of the few studies there are on corticosteroids and lung function after severe COVID-19 [36]. A plausible negative effect of corticosteroid treatment is more frequent VAP which also has been shown to increase the mortality rate [29]. Another possible negative effect of corticosteroid treatment may be delayed viral clearance, which previous studies of corticosteroid treatment in Severe Acute Respiratory Syndrome (SARS) and Middle East Respiratory Syndrome (MERS) coronavirus outbreaks have shown [37, 38]. However, viral load has shown to be highest during the first week of disease onset and subsequently decline and corticosteroids are supposedly introduced after the viral replication has peaked [39]. These findings show the lack of knowledge of the long-term outcomes regarding lung function and therefore, further studies are warranted.

For health-related quality of life outcomes, there was no statistically significant difference between the groups. This may be due to that the ICU survivors in their daily life did not use the maximum of their lung capacity. These results may also suggest that factors other than the extent of lung function are involved in patients’ trajectory of recovery after severe COVID-19 infection, such as neuropsychiatric symptoms (memory and concentration), or mental health problems [40].

### Strength and limitations

The main methodological strengths of the study lie in the comprehensive data collection together with the use of well-validated outcome tests and questionnaires. The study has some limitations. Firstly, this study only included survivors and it is possible that more severely ill patients survived later in the pandemic and there is the risk of selection bias as some COVID-19 survivors opted out of the follow-up program. However, patient characteristics and ICU-related data were similar between survivors who attended the follow-up and non-participants, as presented in a previous study [41]. Further, there were cases with missing information about SAPS 3, but when analyzing the cases with complete data with adjustment for SAPS 3, similar results for pulmonary outcomes where shown. The lack of baseline data prevents the identification of new-onset problems since data on pulmonary function and HRQL is difficult to obtain pre-ICU. The HRQL results were self-reported and may include a risk of response shift. Results were adjusted for known confounders. However, since data on virus strain were not available, we could not adjust for this parameter and residual confounding cannot be ruled out.

## Conclusions

This study demonstrated poorer pulmonary outcomes for COVID-19 intensive care survivors who received corticosteroid treatment compared to those who did not. However, the impact on HRQL seemed to be minor. The results of this study need to be confirmed in future studies.

## Electronic supplementary material

Below is the link to the electronic supplementary material.

Supplementary Material 1

## Data Availability

The datasets used and/or analyzed during the current study are available from the corresponding author upon reasonable request.

## Abbreviations

ARDS: Acute respiratory distress syndrome
BMI: Body mass index
CI: Confidence intervals
DLCO: Diffusion capacity for carbon monoxide
FVC: Forced vital capacity
HFNO: High-flow treatment nasal oxygen
HRQL: Health-related quality of life
ICU: Intensive care unit
IQR: Interquartile range
Md: Median
MERS: Middle East Respiratory Syndrome
MSD: Mean score differences
NIV: Non-invasive ventilation
SAPS 3: Simplified Acute Physiological Score III
SARS: Severe Acute Respiratory Syndrome
sCAP: severe community-acquired pneumonia
IQR: Interquartile range
VAP: Ventilator-associated pneumonia

## References

[1] Batah SS, Fabro AT. Pulmonary pathology of ARDS in COVID-19: A pathological review for clinicians. Respir Med. 2021;176:106239. 33246294 10.1016/j.rmed.2020.106239 PMC7674971

[2] Group WHOREAC-TW, Sterne JAC, Murthy S, Diaz JV, Slutsky AS, Villar J, et al. Association between administration of systemic corticosteroids and mortality among critically ill patients with COVID-19: A Meta-analysis. JAMA. 2020;324(13):1330–41. 32876694 10.1001/jama.2020.17023 PMC7489434

[3] Wagner C, Griesel M, Mikolajewska A, Metzendorf MI, Fischer AL, Stegemann M, et al. Systemic corticosteroids for the treatment of COVID-19: Equity-related analyses and update on evidence. Cochrane Database Syst Rev. 2022;11(11):CD014963. 36385229 10.1002/14651858.CD014963.pub2 PMC9670242

[4] Nhean S, Varela ME, Nguyen YN, Juarez A, Huynh T, Udeh D, et al. COVID-19: A review of potential treatments (Corticosteroids, Remdesivir, Tocilizumab, Bamlanivimab/Etesevimab, and Casirivimab/Imdevimab) and Pharmacological considerations. J Pharm Pract. 2023;36(2):407–17. 34597525 10.1177/08971900211048139 PMC10064180

[5] Hanna G, Bankler S, Schandl A, Roël M, Hedman A, Andersson Franko M, et al. The role of ventilatory support for long-term outcomes after critical infection with COVID-19: A prospective cohort study. Clin Respir J. 2022;16(1):63–71. 34665518 10.1111/crj.13453 PMC8652938

[6] Schandl A, Hedman A, Lynga P, Fathi Tachinabad S, Svefors J, Roel M, et al. Long-term consequences in critically ill COVID-19 patients: A prospective cohort study. Acta Anaesthesiol Scand. 2021;65(9):1285–92. 34097753 10.1111/aas.13939 PMC8212104

[7] Huang C, Huang L, Wang Y, Li X, Ren L, Gu X, et al. 6-month consequences of COVID-19 in patients discharged from hospital: a cohort study. Lancet. 2021;397(10270):220–32. 33428867 10.1016/S0140-6736(20)32656-8 PMC7833295

[8] Jacobson KB, Rao M, Bonilla H, Subramanian A, Hack I, Madrigal M, et al. Patients with uncomplicated coronavirus disease 2019 (COVID-19) have Long-Term persistent symptoms and functional impairment similar to patients with severe COVID-19: A cautionary Tale during a global pandemic. Clin Infect Dis. 2021;73(3):e826–9. 33624010 10.1093/cid/ciab103 PMC7929039

[9] González J, Benítez ID, Carmona P, Santisteve S, Monge A, Moncusí-Moix A, et al. Pulmonary function and radiologic features in survivors of critical COVID-19: A 3-Month prospective cohort. Chest. 2021;160(1):187–98. 33676998 10.1016/j.chest.2021.02.062 PMC7930807

[10] Pavan M, Bassani D, Sturlese M, Moro S. From the Wuhan-Hu-1 strain to the XD and XE variants: is targeting the SARS-CoV-2 Spike protein still a pharmaceutically relevant option against COVID-19? J Enzyme Inhib Med Chem. 2022;37(1):1704–14. 35695095 10.1080/14756366.2022.2081847 PMC9196651

[11] Carbonell R, Urgeles S, Rodriguez A, Bodi M, Martin-Loeches I, Sole-Violan J, et al. Mortality comparison between the first and second/third waves among 3,795 critical COVID-19 patients with pneumonia admitted to the ICU: A multicentre retrospective cohort study. Lancet Reg Health Eur. 2021;11:100243. 34751263 10.1016/j.lanepe.2021.100243 PMC8566166

[12] Reyes LF, Rodriguez A, Bastidas A, Parra-Tanoux D, Fuentes YV, García-Gallo E, et al. Dexamethasone as risk-factor for ICU-acquired respiratory tract infections in severe COVID-19. J Crit Care. 2022;69:154014. 35217370 10.1016/j.jcrc.2022.154014 PMC8863516

[13] Group RC, Horby P, Lim WS, Emberson JR, Mafham M, Bell JL, et al. Dexamethasone in hospitalized patients with Covid-19. N Engl J Med. 2021;384(8):693–704. 32678530 10.1056/NEJMoa2021436 PMC7383595

[14] Chaudhuri D, Sasaki K, Karkar A, Sharif S, Lewis K, Mammen MJ et al. Corticosteroids in COVID-19 and non-COVID-19 ARDS: a systematic review and meta-analysis. Intensive Care Med. 47. United States2021. pp. 521– 37. 10.1007/s00134-021-06394-2 PMC8054852 33876268

[15] Jayasimhan D, Matthay MA. Corticosteroids in adults with acute respiratory distress syndrome and severe pneumonia. BJA Educ. 2023;23(12):456–63. 38009137 10.1016/j.bjae.2023.08.005 PMC10667747

[16] von Elm E, Altman DG, Egger M, Pocock SJ, Gøtzsche PC, Vandenbroucke JP, et al. The strengthening the reporting of observational studies in epidemiology (STROBE) statement: guidelines for reporting observational studies. J Clin Epidemiol. 2008;61(4):344–9. 18313558 10.1016/j.jclinepi.2007.11.008

[17] Svensson P, Hofmann R, Häbel H, Jernberg T, Nordberg P. Association between cardiometabolic disease and severe COVID-19: a nationwide case-control study of patients requiring invasive mechanical ventilation. BMJ Open. 2021;11(2):e044486. 33597145 10.1136/bmjopen-2020-044486 PMC7893210

[18] Metnitz PG, Moreno RP, Almeida E, Jordan B, Bauer P, Campos RA, et al. SAPS 3–From evaluation of the patient to evaluation of the intensive care unit. Part 1: objectives, methods and cohort description. Intensive Care Med. 2005;31(10):1336–44. 16132893 10.1007/s00134-005-2762-6 PMC1315314

[19] Graham BL, Brusasco V, Burgos F, Cooper BG, Jensen R, Kendrick A et al. 2017 ERS/ATS standards for single-breath carbon monoxide uptake in the lung. Eur Respir J. 2017;49(1). 10.1183/13993003.00016-2016 28049168

[20] Hedenstrom H, Malmberg P, Fridriksson HV. Reference values for lung function tests in men: regression equations with smoking variables. Ups J Med Sci. 1986;91(3):299–310. 3811032 10.3109/03009738609178670

[21] Hedenstrom H, Malmberg P, Agarwal K. Reference values for lung function tests in females. Regression equations with smoking variables. Bull Eur Physiopathol Respir. 1985;21(6):551–7. 4074961

[22] Hays RD, Sherbourne CD, Mazel RM. The RAND 36-Item health survey 1.0. Health Econ. 1993;2(3):217–27. 8275167 10.1002/hec.4730020305

[23] Orwelius L, Nilsson M, Nilsson E, Wenemark M, Walfridsson U, Lundstrom M, et al. The Swedish RAND-36 health Survey - reliability and responsiveness assessed in patient populations using Svensson’s method for paired ordinal data. J Patient Rep Outcomes. 2017;2(1):4. 29757320 10.1186/s41687-018-0030-0 PMC5934928

[24] Ohlsson-Nevo E, Hiyoshi A, Norén P, Möller M, Karlsson J. The Swedish RAND-36: psychometric characteristics and reference data from the Mid-Swed health survey. J Patient Rep Outcomes. 2021;5(1):66. 34347192 10.1186/s41687-021-00331-z PMC8339183

[25] Khoudri I, Ali Zeggwagh A, Abidi K, Madani N, Abouqal R. Measurement properties of the short form 36 and health-related quality of life after intensive care in Morocco. Acta Anaesthesiol Scand. 2007;51(2):189–97. 17261146 10.1111/j.1399-6576.2006.01225.x

[26] Heyland DK, Hopman W, Coo H, Tranmer J, McColl MA. Long-term health-related quality of life in survivors of sepsis. Short form 36: a valid and reliable measure of health-related quality of life. Crit Care Med. 2000;28(11):3599–605. 11098960 10.1097/00003246-200011000-00006

[27] Pellegrino R, Viegi G, Brusasco V, Crapo RO, Burgos F, Casaburi R, et al. Interpretative strategies for lung function tests. Eur Respir J. 2005;26(5):948–68. 16264058 10.1183/09031936.05.00035205

[28] Samsa G, Edelman D, Rothman ML, Williams GR, Lipscomb J, Matchar D. Determining clinically important differences in health status measures: a general approach with illustration to the health utilities index mark II. PharmacoEconomics. 1999;15(2):141–55. 10351188 10.2165/00019053-199915020-00003

[29] Reyes LF, Rodriguez A, Fuentes YV, Duque S, García-Gallo E, Bastidas A, et al. Risk factors for developing ventilator-associated lower respiratory tract infection in patients with severe COVID-19: a multinational, multicentre study, prospective, observational study. Sci Rep. 2023;13(1):6553. 37085552 10.1038/s41598-023-32265-5 PMC10119842

[30] Menendez R, Mendez R, Latorre A, Gonzalez-Jimenez P, Peces-Barba G, Molina M, et al. Residual pulmonary infiltrates, symptoms and diffusion impairment at 1-year after severe COVID-19 infection have different associated factors. J Intern Med. 2023;294(1):69–82. 37038609 10.1111/joim.13642

[31] Barros C, Freire RS, Frota E, Rezende Santos AG, Farias MEL, Rodrigues MGA, et al. Short-Course of Methylprednisolone improves respiratory functional parameters after 120 days in hospitalized COVID-19 patients (Metcovid Trial): A Randomized Clinical Trial. Front Med (Lausanne). 2021;8:758405. 34917633 10.3389/fmed.2021.758405 PMC8669506

[32] Dusart C, Smet J, Chirumberro A, Andre S, Roman A, Claus M et al. Pulmonary functional outcomes at 3 months in critical COVID-19 survivors hospitalized during the first, second, and third pandemic waves. J Clin Med. 2023;12(11). 10.3390/jcm12113712 PMC10253272 37297906

[33] Enright Md P. Office-based DLCO tests help pulmonologists to make important clinical decisions. Respir Investig. 2016;54(5):305–11. 27566377 10.1016/j.resinv.2016.03.006

[34] Isitt C, Sjöholm D, Hergens MP, Granath F, Nauclér P. The early impact of vaccination against SARS-CoV-2 in region Stockholm, Sweden. Vaccine. 2022;40(20):2823–7. 35393149 10.1016/j.vaccine.2022.03.061 PMC8960184

[35] Calabretta D, Martìn-Loeches I, Torres A. New guidelines for severe Community-acquired pneumonia. Semin Respir Crit Care Med. 2024;45(2):274–86. 38428839 10.1055/s-0043-1777797

[36] Tekin A, Domecq JP, Valencia Morales DJ, Surapeneni KM, Zabolotskikh IB, Cartin-Ceba R, et al. Biomarker-Concordant steroid administration in severe coronavirus Disease-2019. J Intensive Care Med. 2023;38(11):1003–14. 37226483 10.1177/08850666231177200

[37] Lee N, Allen Chan KC, Hui DS, Ng EK, Wu A, Chiu RW, et al. Effects of early corticosteroid treatment on plasma SARS-associated coronavirus RNA concentrations in adult patients. J Clin Virol. 2004;31(4):304–9. 15494274 10.1016/j.jcv.2004.07.006 PMC7108318

[38] Arabi YM, Mandourah Y, Al-Hameed F, Sindi AA, Almekhlafi GA, Hussein MA, et al. Corticosteroid therapy for critically ill patients with middle East respiratory syndrome. Am J Respir Crit Care Med. 2018;197(6):757–67. 29161116 10.1164/rccm.201706-1172OC

[39] To KK, Tsang OT, Leung WS, Tam AR, Wu TC, Lung DC, et al. Temporal profiles of viral load in posterior oropharyngeal saliva samples and serum antibody responses during infection by SARS-CoV-2: an observational cohort study. Lancet Infect Dis. 2020;20(5):565–74. 32213337 10.1016/S1473-3099(20)30196-1 PMC7158907

[40] Berentschot JC, Bek LM, Heijenbrok-Kal MH, van Bommel J, Ribbers GM, Aerts J, et al. Long-term health outcomes of COVID-19 in ICU- and non-ICU-treated patients up to 2 years after hospitalization: a longitudinal cohort study (CO-FLOW). J Intensive Care. 2024;12(1):47. 39516956 10.1186/s40560-024-00748-w PMC11546104

[41] Darlington P, Roël M, Cronhjort M, Hanna G, Hedman A, Joelsson-Alm E, et al. Comparing severe COVID-19 outcomes of first and second/third waves: a prospective single-centre cohort study of health-related quality of life and pulmonary outcomes 6 months after infection. BMJ Open. 2023;13(7):e071394. 37460259 10.1136/bmjopen-2022-071394 PMC10357304

