# Supplementary material for: Corticosteroids and long-term pulmonary function after critical illness due to COVID-19– a single-center cohort study
Source: BMC Pulm Med. 2025 Apr 26;25:201. doi: 10.1186/s12890-025-03659-0 (PMC12032655; doi:10.1186/s12890-025-03659-0)
Supplement: Supplementary file 1 — Supplementary Material 1 [file 12890_2025_3659_MOESM1_ESM.docx]

Supplementary Table 1. Other treatments than corticosteroids during the first wave; 1 March 2020- 1 September 2020 and later waves; 2 September 2020 – 1 August 2021.

|  | **First wave** | **Later waves** |
| --- | --- | --- |
| All or almost all patients | Oxygen | Oxygen |
|  | Thrombosis prophylaxis | Thrombosis prophylaxis |
|  | Antibiotics | Antibiotics |
| A minority of patients | chloroquine phosphate | Remdisivir |
|  |  | Tocilizumab |

Supplementary Table 2. Comparison between participants who completed the questionnaires; performed the pulmonary function test; and those who did not.

| Measure | Characteristic | Responder | Non-responder | p-value |
| --- | --- | --- | --- | --- |
| **Pulmonary function test** |  | N=192 | N=57 |  |
|  | Age ≥ 65 | 70 (36) | 14 (25) | 0.10 |
|  | Male | 140 (73) | 45 (79) | 0.36 |
|  | Diabetes | 38 (20) | 14 (25) | 0.44 |
|  | Hypertension/  cardiovascular disease | 96 (50) | 29 (51) | 0.91 |
|  | Chronic lung disease | 39 (20) | 11 (19) | 0.87 |
|  | Mechanical ventilation | 89 (46) | 28 (49) | 0.71 |
| **RAND-36** |  | N=169 | N=80 |  |
|  | Age ≥ 65 | 60 (36) | 24 (30) | 0.39 |
|  | Male | 123 (73) | 62 (78) | 0.43 |
|  | Diabetes | 35 (21) | 17 (21) | 0.92 |
|  | Hypertension/  cardiovascular disease | 90 (53) | 35 (44) | 0.16 |
|  | Chronic lung disease | 35 (21) | 15 (19) | 0.72 |
|  | Mechanical ventilation | 77 (46) | 40 (50) | 0.51 |

Supplementary Table 3. Comparison between those with information about SAPS 3 and those with missing information.

| Characteristic | SAPS 3 exists | Missing value | p-value |
| --- | --- | --- | --- |
|  | N=217 | N=32 |  |
| Age ≥ 65 | 75 (35) | 9 (28) | 0.55 |
| Male | 163 (75) | 22 (69) | 0.52 |
| Diabetes | 47 (22) | 5 (16) | 0.64 |
| Hypertension/  cardiovascular disease | 111 (51) | 14 (42) | 0.46 |
| Chronic lung disease | 43 (20) | 7 (22) | 0.81 |
| Mechanical ventilation | 110 (51) | 7 (22) | <0.05 |

SAPS= Simplified Acute Physiology Score
